# Spontaneous Venous Aneurysm: Report of a Non-traumatic Superficial Venous Aneurysm on the Distal Arm

**DOI:** 10.7759/cureus.2641

**Published:** 2018-05-17

**Authors:** Jacqueline McKesey, Philip R Cohen

**Affiliations:** 1 Department of Dermatology, University of California, San Diego, San Diego, USA

**Keywords:** aneurysm, arm, deep, extremity, superficial, thromboembolism, thrombosis, vascular, vein, venous

## Abstract

Venous aneurysms are benign acquired vascular lesions. A 59-year-old man developed a lesion on his right wrist that would enlarge and flatten depending on whether his arm was dependent or elevated; he had no prior history of trauma to the site. Examination of his wrist revealed a soft, compressible nodule contiguous with venous structures in the area. The history and clinical appearance established the diagnosis of a superficial venous aneurysm. Venous aneurysms typically occur on extremities, either in the superficial or deep venous systems; a prior history of trauma is often elicited. Clinical observation may be appropriate for the management of venous aneurysms; however, symptomatic lesions often require excision. In conclusion, venous aneurysms often appear in adults; trauma may or may not precede their appearance. Asymptomatic lesions may be observed, whereas surgery may be necessary to resolve the condition if the aneurysm is symptomatic.

## Introduction

Venous aneurysms are uncommon benign vascular lesions. Venous aneurysms in the upper extremity are rare and often misdiagnosed as soft tissue masses. They are usually acquired and may be preceded by trauma. Venous aneurysms are associated with the potential risk of thromboembolism; therefore, they require correct diagnosis and treatment— either with surgical excision or close observation [1]. A man who developed a superficial venous aneurysm on his right wrist without a prior history of injury to the site is described and the salient features of this condition are reviewed.

## Case presentation

A 59-year-old man with a history of non-melanoma skin cancer was followed by his dermatologist every six months for skin checks. His prior skin lesions included a basal cell carcinoma on the back, squamous cell carcinomas on the cheek and antihelix, and multiple actinic keratoses. Benign lesions included lipomas on his upper extremities. Four years ago, he developed an asymptomatic lesion on his distal flexor right forearm overlying his wrist. There was no history of trauma to the site.

**Figure 1:**
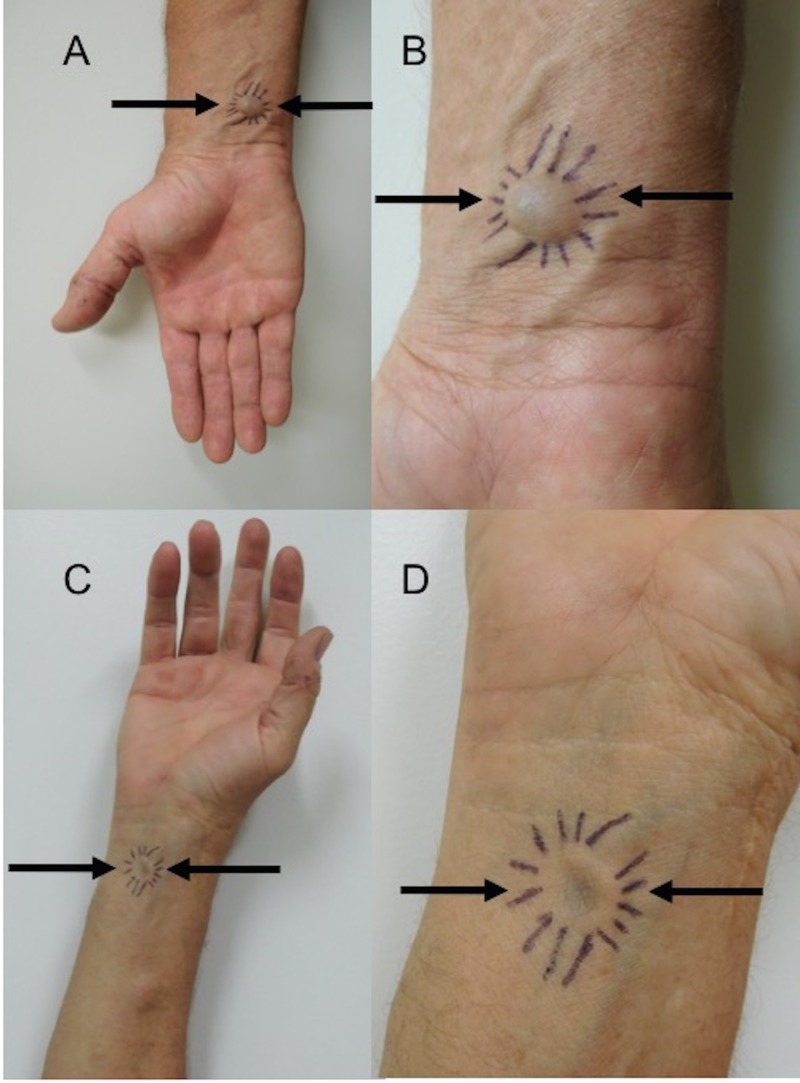
The distal right flexor forearm of a 59-year-old man with a superficial venous aneurysm Distant (A) and closer (B) views show a full aneurysm (arrows) demarcated by the pen marks extending from the nodule; a vein is noted to be entering and exiting the dermal tumor. When the arm is raised, the vascular tumor—distant (C) and closer (D) views— shows flattening of the aneurysm (arrows).

Cutaneous examination of his right forearm showed a soft, 10 x 10 mm, compressible blue subcutaneous nodule that was most prominent when his arm was held in a dependent position (Figure 1). When he would raise his arm above the level of his heart, the nodule would spontaneously flatten.

Correlation of the history and clinical morphology established the diagnosis of a superficial venous aneurysm. The aneurysm did not interfere with the patient’s activities of daily living. Therefore, he declined any additional evaluation and no therapeutic interventions were initiated.

## Discussion

Venous aneurysms are acquired benign vascular lesions. They represent a dilation of a segment of vein that is at least 1.5 times the diameter of the adjacent non-dilated vein. In addition, they are not associated with an arteriovenous communication, pseudoaneurysm, or varicose vein [2].

Venous aneurysms are classified as either superficial or deep. Superficial venous aneurysms occur in the saphenous system of the leg or the basilic and cephalic venous systems of the arm. Deep venous aneurysms involve the femoral venous system of the leg or the deep fascia of the arm [3].

Venous aneurysms are typically described in middle-aged adults. However, they can also occur in childhood or the elderly. They are equally common in men and women. However, they have been observed more frequently in individuals with higher body mass indices [2]. 

Venous aneurysms are typically preceded by trauma; yet, idiopathic lesions may develop. They are usually asymptomatic; however, patients can develop symptoms of vascular reflux including edema, swelling, and pain. Deep venous aneurysms carry a distinction from superficial ones since they have an increased risk of venous thromboembolism [1].

Similar to our patient, superficial venous aneurysms may present as asymptomatic subcutaneous soft nodules that expand and flatten dependent on position of the extremity. In contrast, deep venous aneurysms may present as painful or swollen soft tissue masses. In addition, deep venous aneurysms can also present with deep venous thrombosis or pulmonary embolism [3]. The clinical differential diagnosis for a venous aneurysm includes benign adipose, melanocytic and vascular lesions (Table 1) [4-12].

**Table 1:** Clinical and pathological differential diagnosis of venous aneurysm

Clinical diagnosis	Clinical findings	Pathology findings	Reference
Angiokeratoma	Hyperkeratotic dark red to purple-blue papules, plaques or nodules, commonly seen on the extremities and trunk	Superficial vascular ectasia of the papillary dermis with overlying epidermal hyperkeratosis.	[4]
Arteriovenous malformation	Brown to violaceous plaques that feel warm with a palpable thrill or bruit. Most commonly seen on the head and neck.	Tangle of arteries and veins connected by fistulae that bypass the capillary bed.	[5]
Blue nevus	Dermal papules, plaques or nodules with dark blue or blue-black discoloration. Most commonly seen on the distal extremities, buttocks, scalp and face.	A proliferation of dendritic and spindled melanocytes in the reticular dermis. May be associated with sclerosis.	[6]
Lipoma	Soft, mobile, subcutaneous masses that are commonly seen on the trunk and upper extremities	Mature, white adipose tissue without atypia.	[7]
Varicose vein	Subcutaneous tortuous, dilated veins, typically seen on the legs and worsened with prolonged standing	Vessel wall with hypertrophic smooth muscle layers, disorganized elastin fibers, and increased fibrous tissue.	[8]
Venous aneurysm	Soft compressible blue nodules whose size may vary with valsalva maneuver or position of extremity	Single vessel with fibrous intima and adventitia, loss of smooth muscle, and fragmentation of elastic lamellae.	[9]
Venous lake	Dark blue to violaceous papules commonly found on sun-exposed surfaces of the lips, face, and ears	Single, large dilated vessel with flat endothelial lining and a thin fibrous wall. Thrombus may be present.	[10]
Venous malformation	Blue or purple rubbery nodules that are easily compressible, typically presenting in childhood and growing with age	Dermal or subcutaneous aggregation of multiple, dilated vascular channels with flat endothelial lining; closely packed and often with thrombosis or phleboliths within the vessel lumen.	[11]
Venous pseudoaneurysm	Soft blue mass that may progress over months to years. Commonly iatragenic or congenital.	Dilated vessel with absence of endothelium. Intima and media are thickened.	[12]

Microscopic examination of venous aneurysms typically show thinning or absence of the vascular muscle layer. In addition, fragmentation of elastin fibers and an increase in fibrinous connective tissue may be present [3, 9]. 

Initial evaluation of a venous aneurysm includes venous duplex ultrasound imaging to establish the size as well as the presence of a thrombus. Further diagnostic workup may include computerized tomography and magnetic resonance imaging.

The pathogenesis of venous aneurysms can be idiopathic. Indeed, spontaneous venous aneurysms have been described in congenital vascular syndromes, such as Klippel-Trenaunay-Weber syndrome [1]. Alternatively, venous aneurysms may occur secondary to inflammation, trauma, or venous insufficiency.

A mechanism for venous aneurysm was described in 1951 by Lev and Saphir; they utilized the terms endophlebohypertrophy (congenital) or phlebosclerosis (acquired) [13]. These terms describe the changes in the vessel wall marked initially by proliferation (endophlebohypertrophy) in the first decades of life, followed subsequently by reduction (phlebosclerosis) of smooth muscle, fragmentation of elastin fibers, and increase of fibrous connective tissue with age. This process may be more pronounced in anatomical areas of high vascular trauma, such as the popliteal vein [14].

Complications of untreated venous aneurysms include disfigurement (such as mass or varicosities), edema, pain, thrombophlebitis, or thrombus formation with recurrent thromboembolism [3]. Treatment of venous aneurysm includes either observation or surgical intervention (such as excision or ligation) or, less commonly, endovascular ablation. However, observation may be appropriate for asymptomatic superficial venous aneurysms— similar to our patient.

## Conclusions

Venous aneurysms are benign vascular lesions often preceded by trauma. Superficial venous aneurysms may be diagnosed clinically; their morphology can vary based on the position of the affected extremity. The management of venous aneurysms can include surgical or radiofrequency techniques; however, aneurysms without associated complications may be observed.
